# Acquisition and Longevity of Antibodies to Plasmodium vivax Preerythrocytic Antigens in Western Thailand

**DOI:** 10.1128/CVI.00501-15

**Published:** 2016-02-05

**Authors:** Rhea J. Longley, Arturo Reyes-Sandoval, Eduardo Montoya-Díaz, Susanna Dunachie, Chalermpon Kumpitak, Wang Nguitragool, Ivo Mueller, Jetsumon Sattabongkot

**Affiliations:** a Mahidol Vivax Research Unit, Faculty of Tropical Medicine, Mahidol University, Bangkok, Thailand; b Walter and Eliza Hall Institute of Medical Research, Melbourne, Australia; c Department of Medical Biology, University of Melbourne, Melbourne, Australia; d The Jenner Institute, Nuffield Department of Medicine, The Henry Wellcome Building for Molecular Physiology, University of Oxford, Oxford, United Kingdom; e Mahidol-Oxford Tropical Medicine Research Unit, Mahidol University, Bangkok, Thailand; f Centre for Tropical Medicine, University of Oxford, Oxford, United Kingdom; g Department of Molecular Tropical Medicine and Genetics, Faculty of Tropical Medicine, Mahidol University, Bangkok, Thailand; h ISGlobal, Barcelona Institute for Global Health, Hospital Clínic, Universitat de Barcelona, Barcelona, Spain

## Abstract

Plasmodium vivax is now the dominant Plasmodium species causing malaria in Thailand, yet little is known about naturally acquired immune responses to this parasite in this low-transmission region. The preerythrocytic stage of the P. vivax life cycle is considered an excellent target for a malaria vaccine, and in this study, we assessed the stability of the seropositivity and the magnitude of IgG responses to three different preerythrocytic P. vivax proteins in two groups of adults from a region of western Thailand where malaria is endemic. These individuals were enrolled in a yearlong cohort study, which comprised one group that remained P. vivax free (by quantitative PCR [qPCR] detection, *n* = 31) and another that experienced two or more blood-stage P. vivax infections during the year of follow up (*n* = 31). Despite overall low levels of seropositivity, IgG positivity and magnitude were long-lived over the 1-year period in the absence of qPCR-detectable blood-stage P. vivax infections. In contrast, in the adults with two or more P. vivax infections during the year, IgG positivity was maintained, but the magnitude of the response to P. vivax circumsporozoite protein 210 (CSP210) decreased over time. These findings demonstrate that long-term humoral immunity can develop in low-transmission regions.

## INTRODUCTION

The parasite species Plasmodium vivax is one of the causative agents of the disease malaria. It is the most geographically widespread of the Plasmodium species that cause disease in humans, with an estimated 2.5 billion people currently at risk of infection (1). Clinical disease peaks in children, whereas adults are often parasitemic but asymptomatic (2). In addition, morbidity measures tend to decrease following successive infections (3). These epidemiological observations demonstrate the impact of naturally acquired immunity against P. vivax. Unfortunately, P. vivax has been historically neglected (4), and so we have little understanding of the mechanisms and targets of such immunity.

P. vivax has a complicated life cycle, with stages in human hosts and mosquito vectors. Within humans, injected sporozoites travel to the liver and the first rounds of asexual replication occur within hepatocytes, after which thousands of merozoites are released into the blood stage. The infection of hepatocytes is known as the preerythrocytic stage or liver stage. This stage precedes clinical symptoms and also acts as a bottleneck in the life cycle (before parasite numbers dramatically increase) and, hence, is an attractive target for a malaria vaccine (5). Currently, the most advanced vaccine against Plasmodium falciparum is RTS,S, which was recently given a positive opinion for regulation by the European Medicines Agency. RTS,S is a particulate vaccine targeting the major sporozoite surface protein known as the circumsporozoite protein (CSP) (6) and is speculated to provide protection via antibodies targeting CSP and preventing sporozoite invasion of hepatocytes. Existing anti-CSP antibody titers prior to vaccination were predicted to be an important influence on the postvaccination peak antibody titers (7), demonstrating the need to understand naturally induced antibodies in volunteers in regions where malaria is endemic prior to conducting vaccine trials. Hence, we require a greater understanding of IgG responses to potential P. vivax candidate vaccine antigens in naturally exposed populations.

IgG antibody responses to a number of P. vivax antigens in individuals resident in areas where malaria is endemic have been assessed; however, attention has been focused on blood-stage antigens rather than preerythrocytic antigens (8). As for P. falciparum, CSP is the predominant sporozoite-coating antigen for P. vivax. The thrombospondin-related adhesion protein (TRAP) is another major sporozoite antigen that is important in the motility and invasion of mosquito salivary glands and hepatocytes (9). TRAP has shown promise as a P. falciparum vaccine candidate in humans (10) and as a P. vivax vaccine candidate in mice (11). Another recently identified preerythrocytic antigen expressed on sporozoites is the cell-traversal protein for ookinetes and sporozoites (CelTOS), which is important for the cell traversal of host cells (12). Impressive data in mice indicated cross-species protection with a P. falciparum CelTOS vaccine and challenge with the murine parasite species Plasmodium berghei (13); however, recent evidence has questioned its promise as a vaccine candidate (14). IgG antibody responses to P. vivax CSP have been extensively studied, and they are relatively prevalent in populations in various regions where malaria is endemic (8). To our knowledge, IgG antibody responses to P. vivax TRAP and CelTOS have not been assessed in human populations in areas where malaria is endemic.

The relative longevity of antigen-specific antibody responses to P. vivax is also poorly understood, given that most immunoepidemiological studies conducted have been cross-sectional in design. However, some longitudinal studies have provided evidence that IgG responses to specific blood-stage proteins (i.e., DBP, AMA1, MSP1) can be well maintained for up to 5 months and potentially for 30 years following infection (recently reviewed in reference 8); conversely, for other proteins (or even the same proteins in a different transmission setting) IgG responses have been noted to quickly decline. For P. vivax CSP, relatively well-maintained antibody responses have been identified. In a region of Brazil that suffered an isolated malaria outbreak in 1988, anti-CSP antibody responses were assessed 5 months and 7 years later (15). While both the seropositivity (45% to 20%) and magnitude declined, some individuals were clearly still antibody positive 7 years after exhibiting malarial symptoms. A study in Thailand also identified that 51/159 individuals were able to consistently produce anti-CSP antibodies for 5 months following enrollment (16).

Given the lack of knowledge about naturally acquired immune responses to preerythrocytic antigens, we aimed to determine the stability of IgG responses to P. vivax TRAP and CelTOS in addition to the two major CSP variants, CSP210 and CSP247 (17), in individuals living in a region where malaria is endemic on the Thai-Myanmar border. The availability of well-characterized longitudinal samples from a cohort study conducted in western Thailand from 2013 to 2014 allowed us to assess the longevity of these IgG responses in the presence and absence of blood-stage P. vivax infections. We provide further evidence that IgG responses to P. vivax CSP can be long-lived, even in the absence of reinfection, and we demonstrate that P. vivax TRAP and CelTOS are not substantially immunogenic following exposure in this low-transmission region of western Thailand.

## MATERIALS AND METHODS

### Study population.

A cohort study was undertaken in the Kanchanaburi and Ratchaburi provinces of western Thailand from May 2013 to June 2014. A total of 999 volunteers were enrolled, with ages 1 to 82 years included (median age, 23 years). May represents the beginning of the rainy season in Thailand and roughly the beginning of the peak malaria season. Fourteen active case detection visits were performed over the yearlong cohort (approximately 4 weeks apart). A total of 609 volunteers attended all visits. At each visit, a finger prick blood sample of approximately 250 μl was collected into an EDTA-containing Microtainer (Becton Dickinson and Company), axillary temperature was recorded, and a questionnaire detailing the volunteer's health for the past month was administered (including use of bed net, reported illness, antimalarial drugs taken, and travel history). Two hundred microliters of whole blood was centrifuged at 800 × *g* for 5 min to separate plasma and red blood cells. Genomic DNA was extracted from the blood pellet and was eluted in 50 μl elution buffer using FavorPrep 96-well genomic DNA extraction kits (Favorgen). Four microliters of DNA (equivalent to 8 μl of whole blood) was used in the detection of malaria parasites by quantitative PCR (qPCR) assay using previously published primer and probe sequences (18, 19). Any volunteer with an axillary temperature of ≥37.5°C or with a history of fever between visits was checked for malaria using an SD Bioline Malaria Ag P.f/Pan rapid diagnosis test kit (Standard Diagnostics). Passive case detection for symptomatic illness was conducted at local health clinics within the region. Any confirmed malaria cases were referred to their local health clinics for antimalarial drug treatment. Over the 14 active case detection visits, parasite prevalence ranged from 1.5% to 5% for P. vivax and from 0.2% to 1.4% for P. falciparum (W. Nguitragool, J. Sattabongkot, and I. Mueller, unpublished data).

At the first and last visits of the cohort (approximately 1 year apart, visits 1 and 14), 5-ml venous bleeds were taken from a selection of consenting volunteers (13 to 80 years of age) in the Kanchanaburi province in order to isolate peripheral blood mononuclear cells and to collect larger volumes of plasma. Blood was collected from 498 volunteers at the first visit and from 323 matched volunteers at the last visit. Of the 323 volunteers from whom large volumes of plasma were collected at the first and last time points, 31 volunteers had two or more P. vivax infections (mostly asymptomatic) during the cohort period and attended at least 11 of the 14 visits. These volunteers were selected for antibody analysis along with 31 age- and gender-matched controls who had no detected (P. vivax or P. falciparum) infections during the entire cohort period and who had attended all 14 visits (Table 1). These control volunteers were chosen at random from all of those available. All data and analysis presented in this study relate to this subset of 62 adult volunteers, apart from the control samples described below.

**TABLE 1:** Demographic characteristics of the selected subsets of volunteers

Characteristic	Value for volunteers who were:	
	Exposed	Uninfected
Age	37 (19–63)^*a*^	39 (20–67)
Proportion male	0.71	0.71
% infected (of total visits)^*b*^	50 (14.2–100)	0
No. of visits attended	13 (11–14)	14

a Data are shown as the median, with the range in parentheses when applicable.

b Percentage of visits at which the volunteer was positive by qPCR for Plasmodium vivax blood-stage malaria.

Antibody measurements were also conducted using plasma from two naive controls (one Australian and one Thai, both male) with no known previous exposure to malaria and one historical Thai plasma sample known to be positive to CSP247. Positivity cutoffs were set based on IgG measurements in 21 children (2 to 3 years old, 48% male) from the same cohort. These children had no recorded blood-stage malarial infections (detected by qPCR) at any of the 14 visits of the cohort, and plasma collected by finger prick was used from the second-to-last visit (visit 13). Samples from these children were run singly.

Informed consent and assent (for children aged 7 to ≤13 years) was obtained from all participants in the study, and ethics approval was obtained from the Ethics Committee at the Faculty of Tropical Medicine, Mahidol University (MUTM 2013-027-01). The study was clearly explained to all volunteers.

### Protein expression in HEK-293T cells.

The P. vivax sequences for CSP210 (Belem; GenBank accession number P08677), CSP247 (PNG; GenBank accession number M69059), TRAP (Salvador I; Uniprot accession number A5K806), and CelTOS (Salvador I; Uniprot accession number Q53UB7) were cloned in the expression vector pHLsec (20), which is flanked by the chicken β-actin/rabbit β-globin hybrid promoter with a signal secretion sequence and a Lys-His_6_ tag. Proteins were expressed upon transient transfection in HEK-293T cells with endotoxin-free plasmids in roller bottles (2,125 cm^2^). Secreted proteins were purified from the supernatants by immobilized Ni Sepharose affinity chromatography. The presence of proteins in the elution samples was confirmed using 6×His epitope tag antibody (horseradish peroxidase [HRP] conjugate), monoclonal antibodies (MRA-1028K sporozoite ELISA kit) for CSP210/CSP247, and a horseradish peroxidase-conjugated monoclonal antibody (Invitrogen) in a Western blot. Samples were concentrated using an Amicon Ultra centrifugal filter system (Life Technologies) until reaching 10 ml of final volume. Contaminant proteins and salts were removed from the concentrate by size exclusion purification (SEC) using Superdex medium in the column. Protein concentration after recovery was tested using a Bradford protein assay and purity was assessed by silver staining and by Western blotting (see Fig. S1 to S4 in the supplemental material).

### Measurement of IgG responses.

Antigen-specific antibodies were measured in plasma samples (from the 31 uninfected and 31 exposed volunteers) at visits 1 and 14 using an enzyme-linked immunosorbent assay (ELISA), essentially as previously described (21). Briefly, 96-well flat-bottom MaxiSorp plates (Nunc) were coated with 1 μg/ml antigen in phosphate-buffered saline (PBS) and were incubated overnight at 4°C. Plates were blocked and plasma samples were diluted with PBS containing 0.05% Tween 20 and 5% skim milk. All washing steps were performed using PBS containing 0.05% Tween 20 and using an automated microplate washer (HydroFlex; Tecan). Plasma samples were run in duplicate (with individual replicates on separate plates) at a 1/100 dilution, and two naive plasma samples and one CSP247-positive plasma sample were included as controls on each plate. To measure total IgG, horseradish peroxidase-conjugated goat anti-human IgG (H+L) (Thermo Scientific Pierce) was used at 100 ng/ml. ABTS [2,2′-azinobis(3-ethylbenzothiazoline-6-sulfonic acid)-diammonium salt; Merck Millipore] was used as the substrate, with the optical density (OD) measured after 30 min at 405 nm. Background values from wells with no plasma were subtracted on a per-plate basis. On each plate, control wells containing no antigen (coated only with PBS) were included for each individual plasma sample; the value for each of these wells was subtracted from that for the corresponding well containing antigen. The mean of the duplicates after background and no-antigen control subtraction was then used as the final value. When the coefficient of variation was >20%, the result was repeated (unless the two values were below the positivity cutoff). For each volunteer, samples from each time point were run on the same plate to allow direct comparability.

Positive cutoff values for each protein were created using relevant data from the 21 uninfected children analyzed from the same cohort who experienced no qPCR-detectable malaria infections during the yearlong follow-up period (see Fig. S5 in the supplemental material). The assumption was made that these children were young enough that it was unlikely that they would have had previous unreported malaria infections before the cohort began but that they were old enough that maternal antibodies would no longer be circulating (22). Cutoff background levels were defined as the average plus two times the standard deviation and were set at the following OD values: P. vivax CSP247, 0.68; CSP210, 0.16; TRAP, 0.14; and CelTOS, 0.12.

### Statistical analysis.

As the data were not normally distributed, nonparametric tests were used. The change in OD value from visit 1 to visit 14 was assessed using the Wilcoxon matched-pairs signed-rank test. Statistical difference between groups was assessed using the Mann-Whitney test. Statistical difference in the rates of seropositivity between exposed individuals with or without a current infection was assessed using Fisher's exact test. All statistical analysis was performed in Prism version 6 (GraphPad).

## RESULTS

### Effect of current P. vivax blood-stage infections on IgG positivity and magnitude.

**FIG 1:**
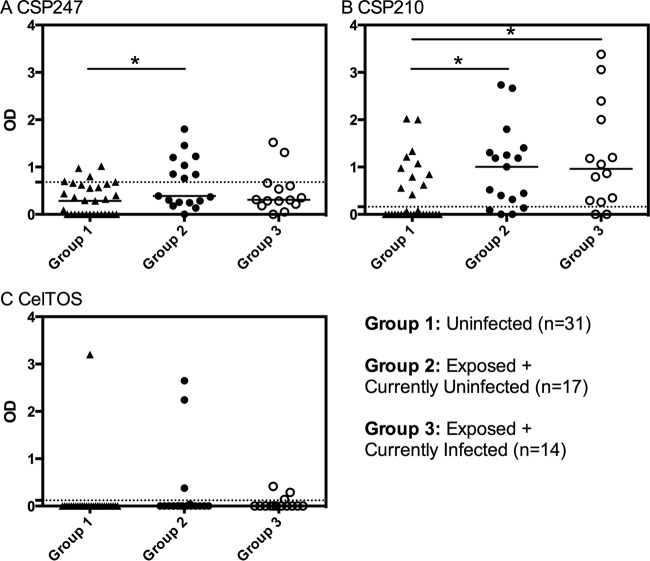
IgG magnitude and the effect of current blood-stage P. vivax infections. IgG responses to P. vivax (A) CSP247, (B) CSP210, and (C) CelTOS at visit 1. Volunteers are stratified into those uninfected throughout the cohort (group 1), those exposed but currently uninfected (group 2), and those exposed but currently infected (group 3). The dashed lines represent protein-specific positivity cutoffs. Statistical differences between the three groups were assessed using the Kruskal-Wallis test with Dunn's multiple-comparison test. *, *P* < 0.05.

We first assessed the effect of current P. vivax blood-stage infections on the IgG response in our exposed group of volunteers to determine whether we could treat these individuals as one group or not. Fourteen of the 31 exposed individuals had a current blood-stage infection at visit 1, and 10 of the 31 had a current blood-stage infection at visit 14. There was no significant difference between individuals with or without a current blood-stage infection in the percentage of volunteers classed as seropositive for CSP210 at either visit, and the percentages were also very similar for CSP247 at visit 14 and for CelTOS at visit 1 (Table 2). A larger (but not significant) percentage of the exposed but currently uninfected volunteers was seropositive for CSP247 at visit 1 (Fisher's exact test, *P* = 0.068), and the opposite was true for CelTOS at visit 14 (*P* = 0.17). There was no detected seropositivity to P. vivax TRAP in either the uninfected or the exposed groups, so responses to this antigen were not included in further analysis. Overall, current blood-stage infections thus appeared to have limited impact on seropositivity. In addition, current blood-stage infections also had little impact on the IgG magnitude of exposed volunteers at visit 1 (Fig. 1) and at visit 14 (see Fig. S6 in the supplemental material). The decision was therefore made to treat all exposed individuals as one group, regardless of the presence or absence of current infections at the time of IgG measurement.

**TABLE 2:** Percentage of volunteers who were seropositive for each antigen at each visit

Antigen	% uninfected (*n* = 31)^*a*^		% exposed (*n* = 31)^*a*^					
			Currently uninfected		Currently infected		Overall	
	V1	V14	V1 (*n* = 17)	V14 (*n* = 21)	V1 (*n* = 14)	V14 (*n* = 10)	V1 (*n* = 31)	V14 (*n* = 31)
CSP210	35	35	76	81	86	80	81	81
CSP247	16	29	47	33	14	30	32	32
CelTOS	3	3	18	14	21	40	19	23
TRAP	0	0	0	0	0	0	0	0

a V1, visit 1; V14, visit 14.

**FIG 2:**
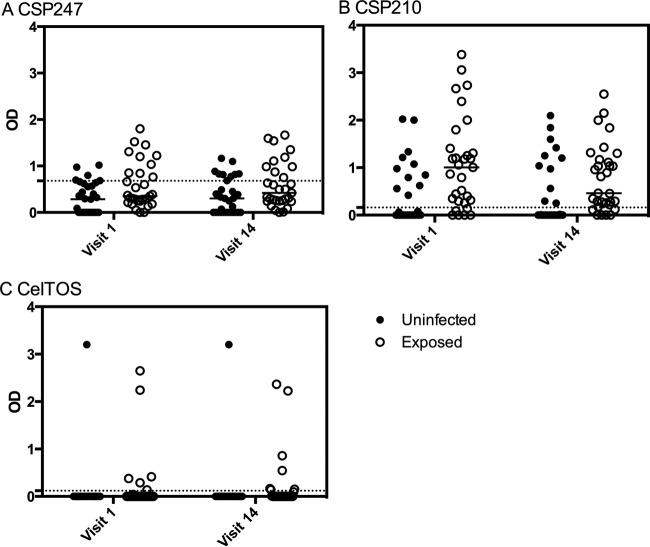
Magnitude of the IgG response and exposure to P. vivax infection. IgG responses to P. vivax proteins at visits 1 and 14 were stratified by the 31 adults who experienced no P. vivax infections (uninfected) and by the 31 adults who experienced 2 or more qPCR-detectable blood-stage P. vivax infections (exposed) during the 1-year cohort. Individual data points and the median are shown. The dashed lines represent protein-specific positivity cutoffs. Statistical difference between uninfected and exposed adults over the two time points was assessed using a two-way ANOVA: (A) *P* = 0.019, (B) *P* = 0.0047, and (C) *P* = 0.5.

Overall, in exposed and uninfected volunteers, seropositivity was highest for P. vivax CSP210 followed by CSP247 and then CelTOS (Table 2). The magnitude of the IgG response was significantly higher in the exposed group of volunteers over the two time points for P. vivax CSP210 and CSP247 (two-way analysis of variance [ANOVA], *P* = 0.0047 and *P* = 0.019, respectively) (Fig. 2) but not for CelTOS, which is likely due to the small number of seropositive individuals (*P* = 0.5). The breadth of the response was also greater in exposed individuals (see Fig. S7 in the supplemental material).

### Stability of the prevalence and magnitude of the IgG responses.

**FIG 3:**
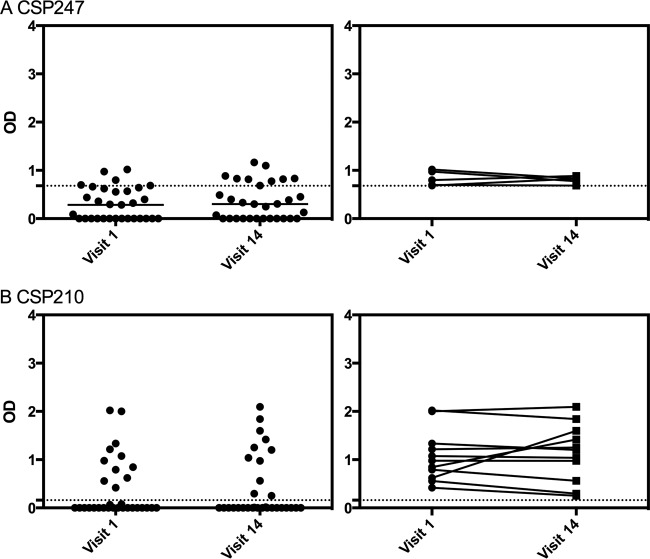
Longevity of the IgG magnitude in uninfected volunteers. IgG responses to P. vivax (A) CSP247 and (B) CSP210 in the 31 uninfected volunteers were stratified by visits 1 and 14. The left panels show individual data points and the median, while the right panels show the individual change over time for individuals who were seropositive. The dashed lines represent protein-specific positivity cutoffs. Statistical difference over visits was assessed using the Wilcoxon matched-pairs signed-rank test: (A) *P* = 0.26, and (B) *P* = 0.46.

**FIG 4:**
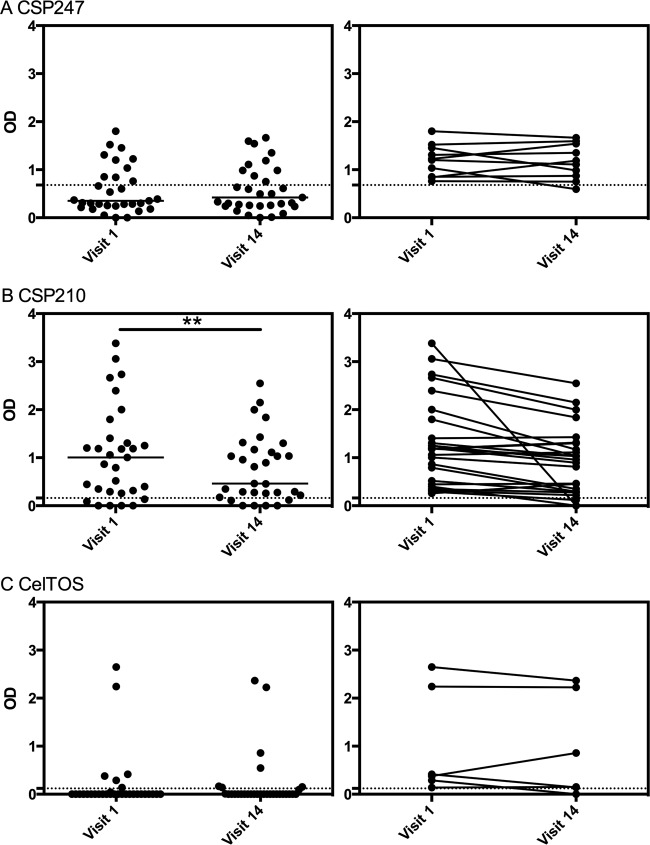
Longevity of the IgG magnitude in exposed volunteers. IgG responses to P. vivax (A) CSP247, (B) CSP210, and (C) CelTOS in the 31 exposed volunteers were stratified by visits 1 and 14. The left panels show individual data points and the median, while the right panels show the individual change over time for individuals who were seropositive. The dashed lines represent protein-specific positivity cutoffs. Statistical difference over visits was assessed using the Wilcoxon matched-pairs signed-rank test: (A) *P* > 0.99, (B) *P* = 0.0014, and (C) *P* = 0.77.

We next assessed the longevity of IgG positivity and magnitude over the yearlong cohort period and how this was affected by the presence or absence of blood-stage infections. Overall, there was very little change in seropositivity status over the year, whether the volunteers were exposed or not to blood-stage infections (Table 3). There was also no significant difference between visits 1 and 14 in the magnitudes of the IgG responses to P. vivax CSP247 or CSP210 in the uninfected group of volunteers (Wilcoxon matched-pairs signed-rank test, *P* = 0.26 and *P* = 0.46, respectively) (Fig. 3), which suggests that ongoing qPCR-detectable P. vivax blood-stage infections are not required to maintain antibody responses to these preerythrocytic proteins. P. vivax CelTOS was not assessed given the limited seropositivity in uninfected individuals. Similarly, there was no significant difference between visits 1 and 14 in the magnitudes of the IgG responses to P. vivax CSP247 and CelTOS in the exposed group of volunteers (*P* > 0.99 and *P* = 0.77, respectively) (Fig. 4); however, for P. vivax CSP210, the magnitude of the IgG response significantly declined from visit 1 to visit 14 for the exposed volunteers (*P* = 0.0056) (Fig. 4).

**TABLE 3:** Percentage of volunteers who maintained seronegativity or seroconverted and who maintained seropositivity or seroreverted

Antigen	Maintained seronegativity (%)		Seroconverted (%)		Maintained seropositivity (%)		Seroreverted (%)	
	Uninfected	Exposed	Uninfected	Exposed	Uninfected	Exposed	Uninfected	Exposed
CSP210	100	50	0	50	100	88	0	12
CSP247	85	95	15	5	100	90	0	10
CelTOS	100	92	0	8	100	83	0	17
TRAP	100	100	0	0	NA^*a*^	NA	NA	NA

a NA, not applicable.

## DISCUSSION

The current study presents new insights into the acquisition and stability of IgG responses to preerythrocytic P. vivax proteins in a low-transmission region. Overall, seropositivity to P. vivax CSP210 was highest, followed by CSP247 and then CelTOS, in both uninfected and exposed individuals, with similar percentages 1 year later. The higher prevalence of IgG responses to CSP210 over those to CSP247 is consistent with the predominance of CSP210 in previous genotyping of P. vivax parasites in this region (23). The lack of IgG responses to P. vivax TRAP is consistent with the absence of or low responses to P. falciparum TRAP in low to moderate transmission regions (24–26). To our knowledge, this is the first time IgG responses have been assessed to P. vivax TRAP in naturally exposed volunteers. In addition, we report the first evidence of naturally induced IgG responses to P. vivax CelTOS in human volunteers. We found relatively higher and more prevalent IgG responses in volunteers exposed to P. vivax blood-stage parasites, as well as a greater breadth of response, at the beginning and end of the 1-year cohort, which indicates that these IgG responses are most likely reflective of long-term exposure and risk of malaria rather than of recent exposure.

Antibody responses to P. vivax CSP have been extensively studied in the past (8); however, the majority of these studies have been cross-sectional, resulting in limited data concerning the longevity and stability of responses. We have shown that IgG positivity to P. vivax CSP210 and to P. vivax CSP247 was well maintained over a 1-year period even in the absence of qPCR-detected blood-stage infections. This builds on previous evidence of antibody longevity in the absence of detectable exposure to P. vivax CSP (15, 16) and to other proteins in a similar low-transmission setting: Wipasa et al. identified long-lived IgG responses to three P. vivax blood-stage proteins in northern Thailand (27). This suggests that either memory B cells or long-lived plasma cells were induced to provide an ongoing source of measurable IgG. Whether or not specific antigen stimulation is required for differentiation of memory B cells into antibody-secreting cells is still contentious (28, 29). There has been speculation that the presence of hypnozoites may provide antigenic stimulation in the absence of new P. vivax infections (30); however, given that the relapse period for Southeast Asia is of the frequent-relapse phenotype (31), it seems unlikely that these individuals (who had no qPCR-detectable blood-stage infections for 1 year) harbored the dormant liver stages. Another potential source of antigenic stimulation may be failed P. vivax infections, i.e., where sporozoites were inoculated but failed to establish (qPCR-detectable) blood-stage infections. To tease out the potential mechanism of such long-lived antibodies, antigen-specific B cell phenotyping will be ultimately required.

Conversely, we noted a statistically significant, albeit small, decrease in the magnitude of the IgG response to P. vivax CSP210 over the yearlong period in individuals who experienced two or more qPCR-detectable blood-stage P. vivax infections. Plasma samples from these individuals were run as matched pairs on the same ELISA plate and, hence, are directly comparable over time. However, a limitation of our study is that only two time points were assessed, so we do not know how the IgG magnitude fluctuated over the yearlong period. It is also important to note that the presence of blood-stage P. vivax does not necessarily indicate a new infection, as it may be due to a relapse. Hence, one plausible explanation is that the most recent preerythrocytic exposure was to sporozoites expressing CSP210, leading to a boosting of the IgG response that then underwent an initial rapid decay followed by a maintenance phase of long-lived IgG (32). In this setting, it is unknown what proportion of detected blood-stage infections is due to hypnozoite activation rather than new infections, but modeling (33) and a field trial in Papua New Guinea suggest it may be very large (34). We would also expect short- and long-lived plasma cells to be contributing to the IgG magnitude in our exposed volunteers, creating more variability in the response. An alternate explanation for the reduced IgG magnitude of P. vivax CSP210 antibodies in individuals with multiple blood-stage P. vivax infections that cannot yet be ruled out is the presence of regulatory T cells (Tregs) (35) or atypical B cells, both of which can suppress B cell Ig production (36, 37). However, it must be noted that some of the exposed individuals had only two qPCR-detected blood-stage infections, and this may not be enough to induce suppression of the IgG magnitude by Tregs or atypical memory B cells. It will be important to determine whether the decrease in the IgG response seen is replicated in a larger sample size and to elucidate the mechanism in order to determine whether relapsing and/or new infections can have a detrimental impact on vaccine efficacy (if a CSP-based vaccine was introduced into this region).

In conclusion, despite the simple immunoassay we employed, our well-defined samples from a carefully designed epidemiological cohort study have provided interesting data and insights into the longevity of antigen-specific IgG responses to preerythrocytic P. vivax antigens in a low-transmission setting. Our findings have raised a number of questions about the effect of antigenic exposure on the development and maintenance of long-lived IgG responses, and hence we propose the following directions for future research: phenotypic examination of (i) memory B cells, (ii) Tregs, and (iii) atypical memory B cells in a similar cohort of volunteers where peripheral blood mononuclear cells are available in addition to plasma samples. It will also be important to consider what effect such preexisting IgG responses to P. vivax CSP might have on a vaccine targeting this antigen. Based on data from P. falciparum CSP (7), in adults the presence of an IgG response to P. vivax CSP might enhance the IgG response induced by a vaccine, and importantly we have demonstrated that these responses have the potential to be long-lived even in the absence of boosting infections. Furthermore, while requiring further study with a larger sample size, our results suggest that IgG responses to P. vivax CSP may be able to identify individuals at a higher risk of malaria in this region. It will be of interest to include these data in the overall analysis currently being conducted on this cohort study alongside the behavioral risk factors.

## Supplementary Material

Supplemental material
