# Supplementary material for: Acquisition and Longevity of Antibodies to Plasmodium vivax Preerythrocytic Antigens in Western Thailand
Source: Clin Vaccine Immunol. 2016 Feb 5;23(2):117–24. doi: 10.1128/CVI.00501-15 (PMC4744911; doi:10.1128/CVI.00501-15)
Supplement: Supplemental material [file supp_23_2_117__index.html]

Acquisition and Longevity of Antibodies to Plasmodium vivax Preerythrocytic Antigens in Western Thailand — Supplemental material 

# Acquisition and Longevity of Antibodies to Plasmodium vivax Preerythrocytic Antigens in Western Thailand

## Supplemental material

- Supplemental file 1 -

  Fig. S1. Silver staining and Western blot analyses of *P. vivax* CSP247 protein after SEC purification. Fig. S2. Silver staining and Western blot analyses of *P. vivax* CSP210 protein after SEC purification. Fig. S3. Silver staining and Western blot analyses of *P. vivax* CelTOS protein after SEC purification. Fig. S4. Silver staining and Western blot analyses of *P. vivax* TRAP protein after SEC purification. Fig. S5. IgG responses to *P. vivax* proteins CSP247, CSP210, TRAP, and CelTOS in 21 uninfected children from western Thailand. Fig. S6. IgG magnitude and the effect of current blood-stage *P. vivax* infections. Fig. S7. Breadth of the IgG response.

  PDF, 1.9M
