# Supplementary material for: Acquisition and Longevity of Antibodies to Plasmodium vivax Preerythrocytic Antigens in Western Thailand
Source: Clin Vaccine Immunol. 2016 Feb 5;23(2):117–24. doi: 10.1128/CVI.00501-15 (PMC4744911; doi:10.1128/CVI.00501-15)
Supplement: Supplemental material [file CVI.00501-15_zcd999095301so1.pdf]

SUPPLEMENTAL MATERIAL

SUPPLEMENTAL FIGURES

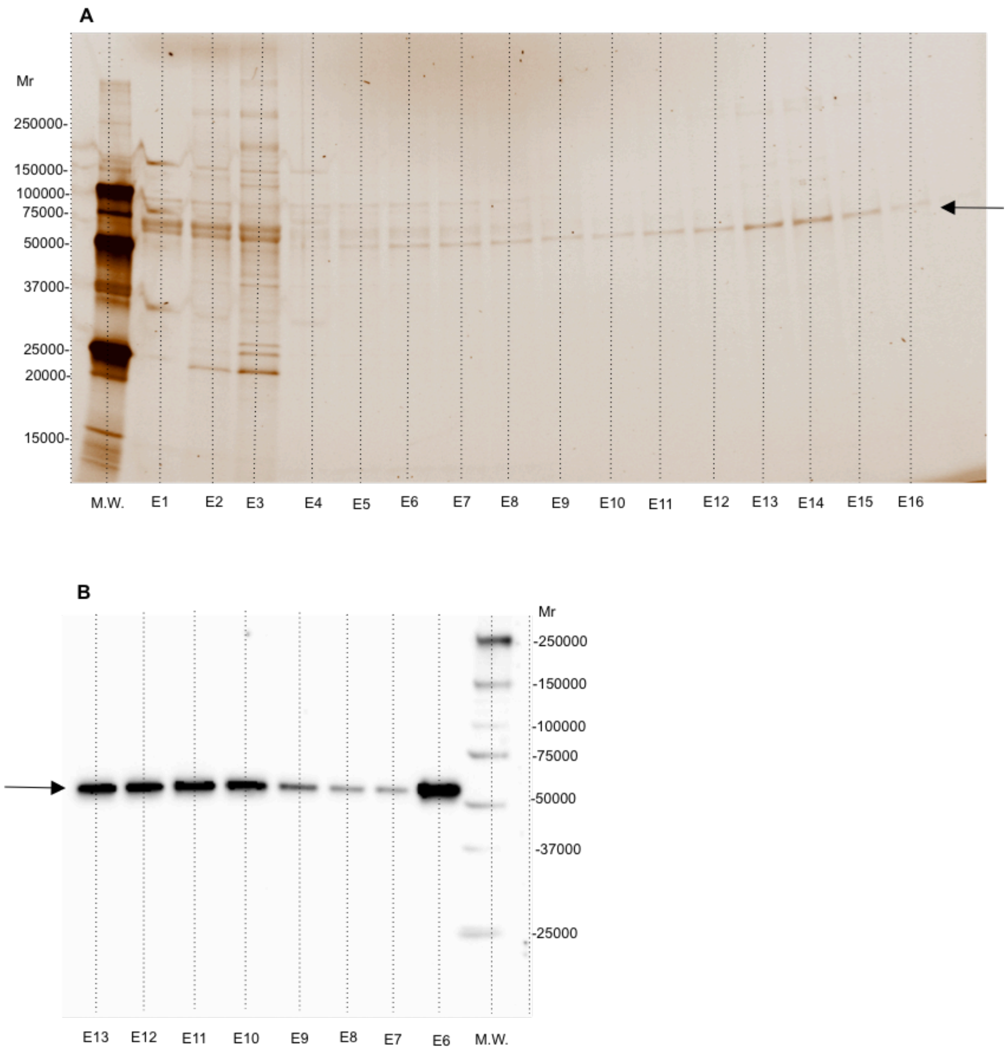

FIG S1 Silver staining and western blot analyses of *P. vivax* CSP247 protein after SEC purification. (A) Elution samples after SEC purification were submitted to 12.5% SDS-PAGE under reducing conditions and stained with silver stain. E6 to E13 elution fractions were selected for western blot analyses (B) using a monoclonal antibody (MRA-1028K sporozoite ELISA kit, Malaria Research and Reference Reagent Resource Center (MRA4)) for CSP247. The expected size of *P. vivax* CSP247 was 50 kDa and was estimated by comparison with known molecular weight standards (Lane 1). These elution samples were concentrated for the ELISA assay.

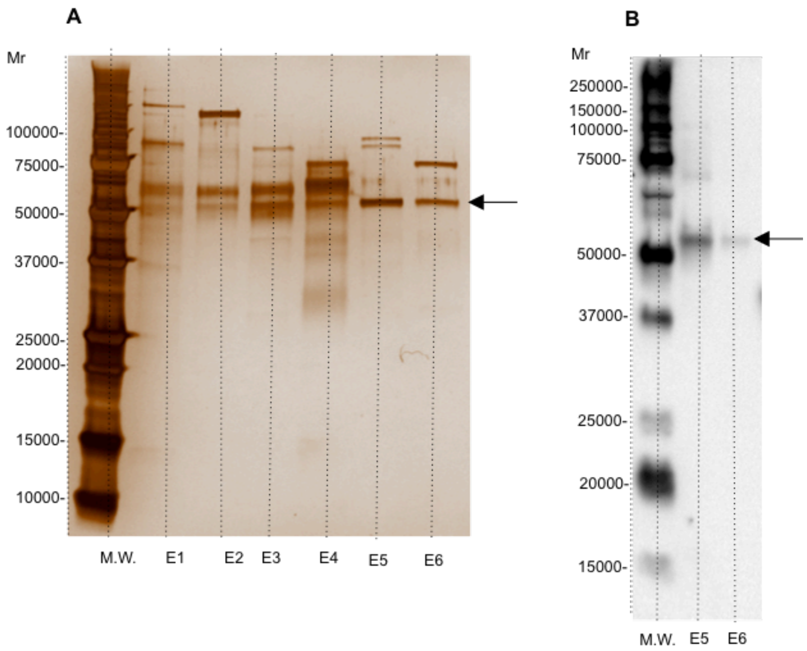

FIG S2 Silver staining and western blot analyses of *P. vivax* CSP210 protein after SEC purification. (A) Elution samples after SEC purification were submitted to 12.5% SDS-PAGE under reducing conditions and stained with silver stain. E5 and E6 elution fractions were selected for western blot analyses (B) using a monoclonal antibody (MRA-1028K sporozoite ELISA kit, Malaria Research and Reference Reagent Resource Center (MRA4)) for CSP210. The expected size of *P. vivax* CSP210 was 50 kDa and was estimated by comparison with known molecular weight standards (Lane 1). These elution samples were concentrated for the ELISA assay.

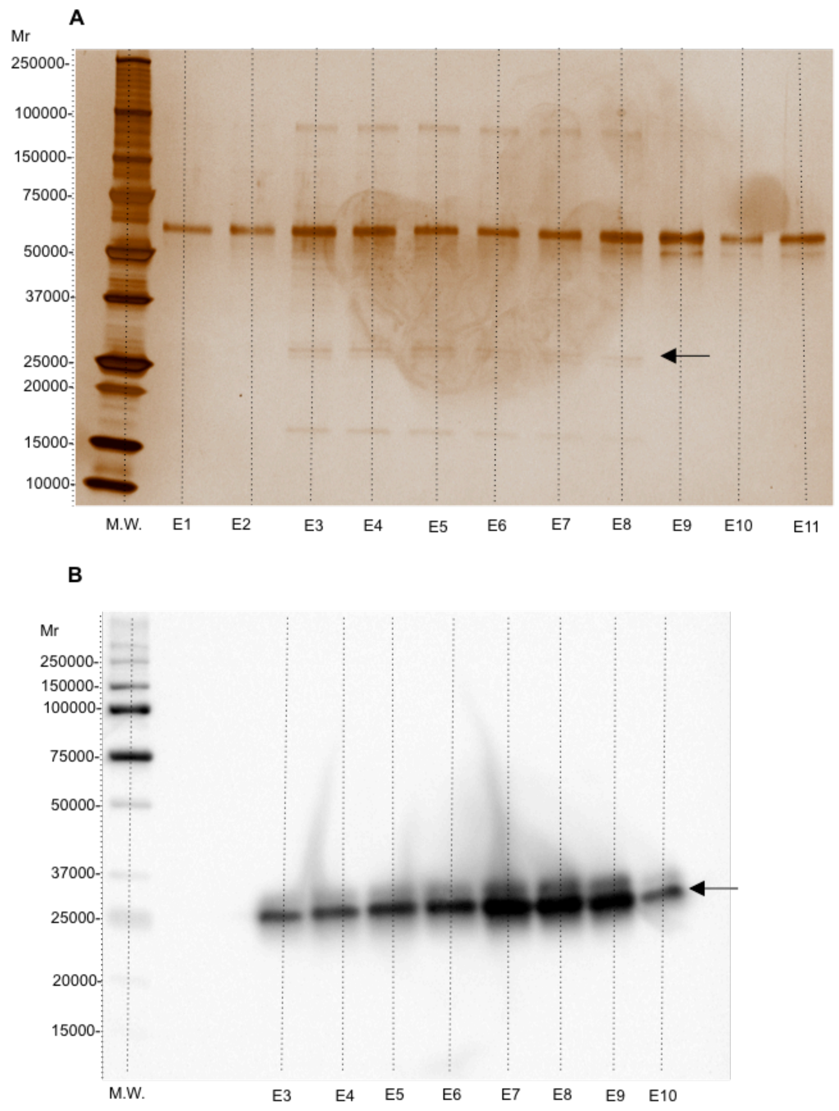

FIG S3 Silver staining and western blot analyses of *P. vivax* CelTOS protein after SEC purification. (A) Elution samples after SEC purification were submitted to 12.5% SDS-PAGE under reducing conditions and stained with silver stain. E3 to E10 elution fractions were selected for western blot analyses (B) using a 6X-His Epitope tag horsedish peroxidase conjugated monoclonal antibody. The expected size of *P. vivax* CelTOS was 30 kDa and was estimated by comparison with known molecular weight standards (Lane 1). These elution samples were concentrated for the ELISA assay.

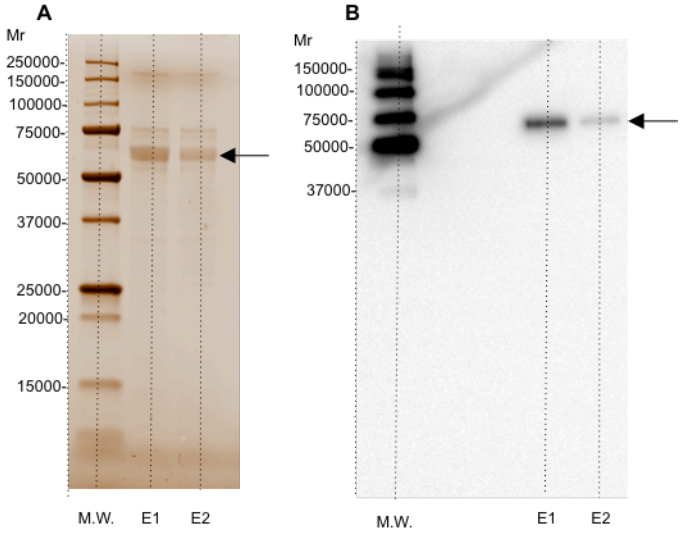

FIG S4 Silver staining and western blot analyses of *P. vivax* TRAP protein after SEC purification. (A) Elution samples after SEC purification were submitted to 12.5% SDS-PAGE under reducing conditions and stained with silver stain. E1 and E2 elution fractions were selected for western blot analyses (B) using a 6X-His Epitope tag horsedish peroxidase conjugated monoclonal antibody. The expected size of *P. vivax* TRAP was 60 kDa and was estimated by comparison with known molecular weight standards (Lane 1). These elution samples were concentrated for the ELISA assay.

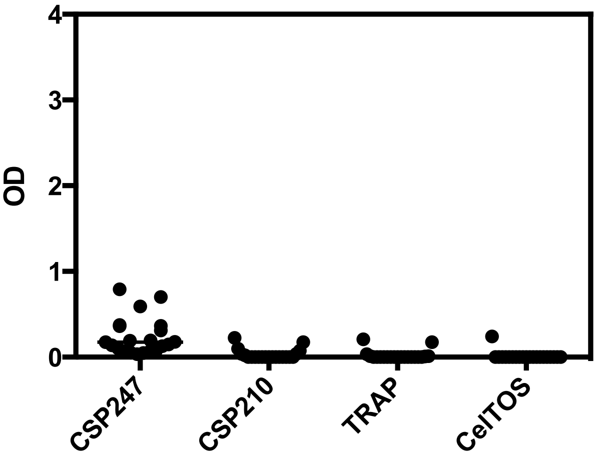

FIG S5 IgG responses to Pv proteins CSP247, CSP210, TRAP and CelTOS in 21 uninfected children from Western Thailand. Individual data points and the median are shown.

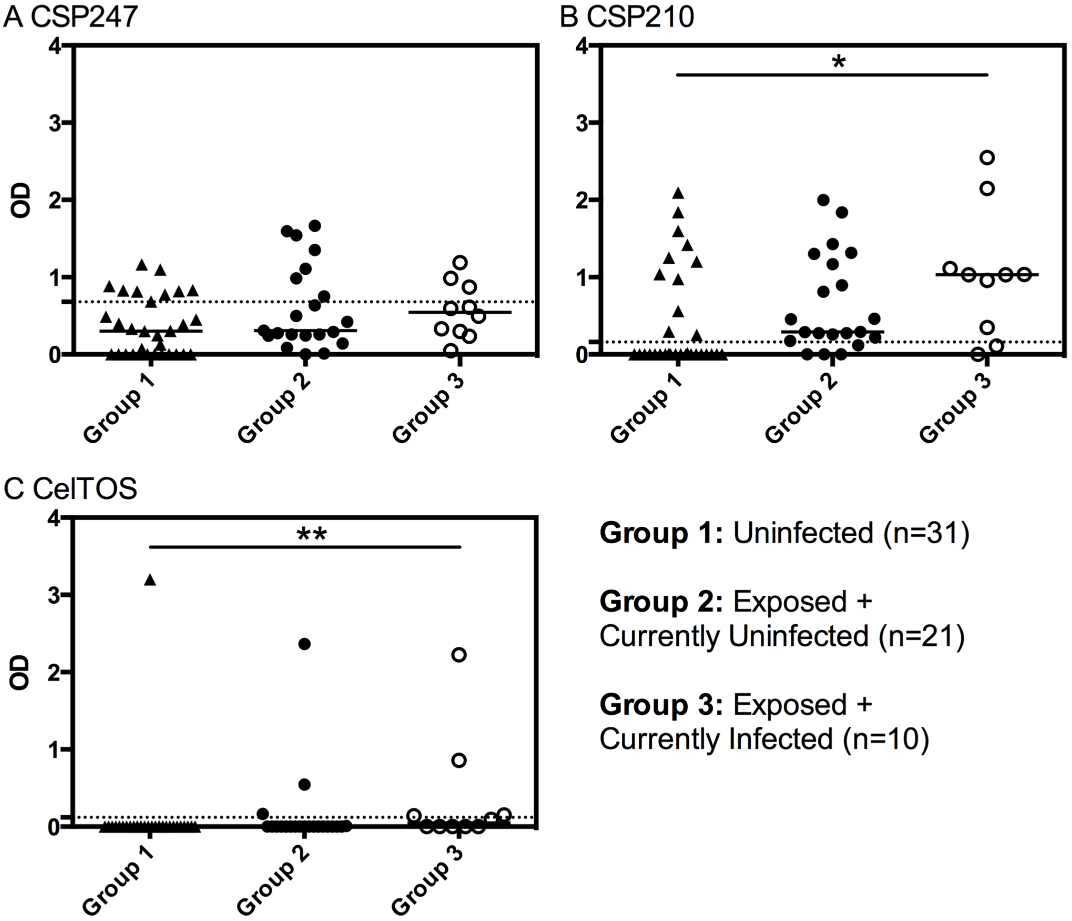

FIG S6 IgG magnitude and the effect of current blood-stage *P. vivax* infections. IgG responses to Pv (A) CSP247 (B) CSP210 and (C) CelTOS at visit 14. Volunteers are stratified into those uninfected throughout the cohort (Group A, n=31), those exposed but currently uninfected (Group B, n=21) and those exposed but currently infected (Group C, n=10). The dashed line represents protein-specific positivity cut-offs. Statistical difference between the three groups was assessed using the Kruskal-Wallis test with Dunn's multiple comparisons test. * p<0.05, ** p<0.01.

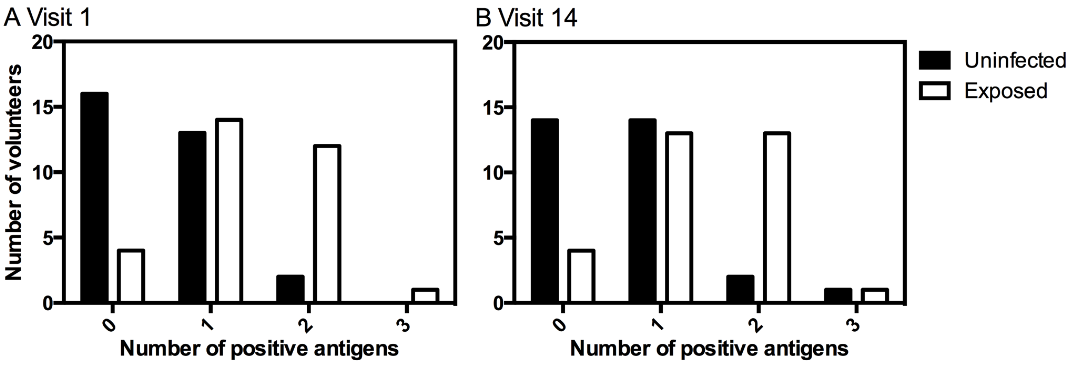

FIG S7 Breadth of the IgG response. The number of Pv proteins each volunteer was IgG positive for after protein-specific positivity cut-offs is shown, stratified by exposure status (n=31 in each group). (A) Visit 1 and (B) Visit 14.
